# fMRI neurofeedback of higher visual areas and perceptual biases

**DOI:** 10.1016/j.neuropsychologia.2016.03.031

**Published:** 2016-05

**Authors:** I. Habes, S. Rushton, S.J. Johnston, M.O. Sokunbi, K. Barawi, M. Brosnan, T. Daly, N. Ihssen, D.E.J. Linden

**Affiliations:** a Cardiff University Brain Research Imaging Centre, School of Psychology, Cardiff University, Cardiff CF10 3AT, UK; b School of Human and Health Sciences, Department of Psychology, Swansea University, Swansea SA2 8PP, UK; c Trinity College Institute of Neuroscience and School of Psychology, Trinity College Dublin, Dublin 2, Ireland; d Cognitive Neuroscience Sector, International School for Advanced Studies (SISSA), 34136 Trieste, Italy; e Neuroscience and Mental Health Research Institute, Cardiff University, Cardiff CF24 4XN, UK

**Keywords:** Neurofeedback, Self-regulation, Parahippocampal place area (PPA), Fusiform face area (FFA), Binocular rivalry

## Abstract

The self-regulation of brain activation via neurofeedback training offers a method to study the relationship between brain areas and perception in a more direct manner than the conventional mapping of brain responses to different types of stimuli. The current proof-of-concept study aimed to demonstrate that healthy volunteers can self-regulate activity in the parahippocampal place area (PPA) over the fusiform face area (FFA). Both areas are involved in higher order visual processing and are activated during the imagery of scenes and faces respectively. Participants (N=9) were required to upregulate PPA relative to FFA activity, and all succeeded at the task, with imagery of scenes being the most commonly reported mental strategy. A control group (N=8) underwent the same imagery and testing procedure, albeit without neurofeedback, in a mock MR scanner to account for any non-specific training effects. The upregulation of PPA activity occurred concurrently with activation of prefrontal and parietal areas, which have been associated with ideation and mental image generation. We tested whether successful upregulation of the PPA relative to FFA had consequences on perception by assessing bistable perception of faces and houses in a binocular rivalry task (before and after the scanning sessions) and categorisation of faces and scenes presented in transparent composite images (during scanning, interleaved with the self-regulation blocks). Contrary to our expectations, upregulation of the PPA did not alter the duration of face or house perception in the rivalry task and response speed and accuracy in the categorisation task. This conclusion was supported by the results of another control experiment (N=10 healthy participants) that involved intensive exposure to category-specific stimuli and did not show any behavioural or perceptual changes. We conclude that differential self-regulation of higher visual areas can be achieved, but that perceptual biases under conditions of stimulus rivalry are relatively robust against such internal modulation of localised brain activity. This study sets the basis for future investigations of perceptual and behavioural consequences of localised self-regulation of neural activity.

## Highlights

• Healthy participants trained differential self-regulation of higher visual areas.
• They were instructed to up-regulate the PPA whilst keeping FFA activity down.
• Up-regulation of the PPA was accompanied by frontal and parietal activation.
• No shift in perceptual biases in scene/face perceptual rivalry tasks was observed.

## 1 Introduction

What is the relationship between activation in a visual area and perception? Real-time (rt)-fMRI-based neurofeedback provides a new way of addressing this question. Neurofeedback enables people to learn to regulate levels or patterns of local brain activation through use of real-time feedback of the blood oxygenation level-dependent (BOLD) signal, provided online in the scanner. Once a participant is able to self-regulate BOLD in a region the relationship between activity and perception can be explored directly. Two previous studies have employed this method to investigate self-regulation of lower visual areas and its perceptual consequences. Scharnowski et al. (2012) investigated whether perceptual enhancements would occur after participants learnt to increase ongoing spontaneous activity at specific retinotopic locations. They found that successful up-regulation resulted in improved detection of a near-threshold visual stimulus presented in the visual field position corresponding to the retinotopic location of the region from which participants had previously received neurofeedback. Shibata et al. (2011) investigated whether successful up-regulation of primary and secondary visual areas could enhance perceptual learning. They used multivariate pattern analysis to identify activation patterns associated with the presentation of Gabor patches of different orientations. The target for the neurofeedback training was to bring ongoing activation as close as possible to the pattern associated with a specific orientation; when the correspondence was high, participants showed improved performance in an orientation discrimination task that was specific to the trained target orientation. These two studies provide a persuasive demonstration of perceptual enhancements caused by self-regulation of activation in lower visual areas.

The potential effects of self-regulation of localised brain activation on perceptual biases has been investigated by Ekanayake et al. (2013) who trained participants to up-regulate either the FFA or the PPA over three scanning sessions and assessed the effects using a binocular rivalry (BR) paradigm. In the BR paradigm, a different image is presented to each eye and both images compete for conscious awareness; participants commonly report periods of seeing one or the other image (rather than a fused image), with spontaneous switches occurring every few seconds, even though the retinal stimulation remains constant during these switches. Ekanayake et al. (2013) found that the non-target percept (that is, a house for those who trained upregulation of the FFA, and a face for those who trained upregulation of the PPA) was perceived for shorter periods not only when neurofeedback was directly coupled with the binocular rivalry task but also afterwards. Such persistence of neurofeedback effects beyond the actual upregulation procedure in the scanner would be particularly relevant for any future clinical applications of such training effects.

Binocular rivalry can be modulated by attention in some circumstances (Dieter and Tadin, 2011, Dieter et al., 2015). The perceptual change induced by Ekanayake et al.’s (2013) neurofeedback protocol was likely mediated by changes in attention (Chong et al., 2005) as participants attempted to focus on the stimulus corresponding to the target area, thereby biasing perception to the target-stimulus and away from the non-target stimulus. In the present study we intended to go beyond Ekanayake et al.’s (2013) study by using differential PPA and FFA self-regulation. Our central research question was whether differential self-regulation is possible and whether it helps to create persistent perceptual biases? We predicted that differential feedback training that involves directing attention to one visual category and away from the other should induce even stronger perceptual biases than upregulation of single areas.

Another aim of the differential feedback protocol was to address the inherent problem associated with single-region feedback that is posed by the limited understanding of the functional relevance of a change in the BOLD signal within a ROI. For example the FFA and PPA are strongly interconnected regions (Stephan et al., 2008), and both receive input from lower visual areas. Thus, if we studied changes in the PPA signal alone, we could not rule out potential modulatory effects from the FFA affecting the BOLD signal from the PPA or effects of lower visual areas driving both PPA and FFA. Furthermore, by employing feedback from multiple regions, we corrected for global drifts and whole brain activation changes that can influence neurofeedback signals obtained from single areas.

As an early example of this procedure, Weiskopf et al. (2004) have shown that it is possible to train participants to differentially self-regulate the supplementary motor area (SMA) and PPA. Robineau et al. (2014) used differential feedback from visual cortex of the left and right hemisphere. They found that eight out of 14 healthy young participants learned to control the signal over the course of three sessions and upregulation of the signal was maintained in a transfer run. However, no perceptual changes, as measured using a visual detection task and a line-bisection task, were observed in those who learned to upregulate the target ROI. No previous study has explored people's ability to regulate the activation difference between visual areas that are selective for different stimulus categories.

In addition to demonstrating the feasibility of differential self-regulation of higher visual areas we expected to obtain perceptual effects, both during and potentially after, the neurofeedback procedure. The possibility of lasting changes in perceptual biases was based on the previous literature. First, as already noted above, Shibata et al., 2011, Ekanayake et al., 2013 found that neurofeedback training can lead to persisting perceptual effects. Second, in our task, it is likely that participants will use mental imagery to regulate activity in FFA and PPA. The broader behavioural literature on mental imagery indicates that performing mental imagery can bias subsequent perception towards the imagined stimulus, for example a particular coloured pattern (Pearson et al., 2008). Therefore it appeared likely that differential up-regulation of PPA over FFA would produce a prolonged bias towards scenes over faces that would be detectable not just during but also immediately after the scanning session. However, to anticipate results, we did not find any change in either of our two tasks (a binocular rivalry task and a semantic judgement task applied to composite face/scene stimuli) although participants successfully up-regulated PPA over FFA activity.

## 2 Materials and methods

### 2.1 Subjects

Seventeen participants were recruited via the participant panel of Cardiff University. All participants had normal or corrected-to-normal vision. Participants were randomly allocated to the experimental or control group. The experimental group (neurofeedback, NF) performed a neurofeedback experiment in the scanner in which they trained to upregulate the activation difference between the PPA and the FFA by category-specific imagery whereas the control group (imagery only, IM) underwent a matched procedure in a mock scanner in which they were instructed to engage in imagery of scenes. Nine participants (5 female, average age=23.4 years) were assigned to the NF group and eight participants (all female, average age=22.6 years) to the IM group. All participants gave written informed consent at the beginning of the study, and the study was approved by the Ethics Committee of the School of Psychology, Cardiff University. Participants were given the choice to receive course credits or payment for their time. There was no significant age difference between the experimental and control groups of the neurofeedback experiment (*p*>.5). Although we did not expect any gender-specific effects on our tasks we tested for any gender imbalances for sake of completeness. There was a significant association between *Group* and *Gender* (χ^2^ [1]=4.650, *p*=.031). Imbalances in the gender distribution can occur in randomised designs with small numbers of participants.

For the perceptual control experiment (see below, Section 2.9), ten healthy (8 females and 2 males, range 21–36 years of age, mean age, 22.9 years) right-handed volunteers with normal vision were recruited from the same student population of Cardiff University as the imaging group. All participants gave written informed consent at the beginning of the study and received course credits for their participation.

### 2.2 Questionnaires

In order to investigate any relation between self-regulation ability and cognitive control, the Thought Control Questionnaire (TCQ; Wells and Davies, 1994) and the Thought Control Ability Questionnaire (TCAQ; Luciano et al., 2005) were administered. As it was unlikely that individuals without the capacity to perform vivid mental imagery would be able to successfully achieve self-regulation, the Vividness of Visual Imagery Questionnaire (VVIQ; Marks, 1973) was used as a screening measure. Only participants with an average score of three or lower were included in the study (lower scores reflecting more vivid imagery) because previous work has shown that those who report more vivid imagery have stronger subsequent perceptual biases (Pearson et al., 2011).

### 2.3 Structural brain imaging procedure

Participants in the NF group underwent structural brain scanning in a 3 T whole body scanner (General Electric, Milwaukee, USA) with an 8-channel head coil. The parameters used for the acquisition of a high-resolution anatomical scan (Fast Spoiled Gradient-Recalled-Echo [FSPGR] sequence) were: 178 slices, TE=3 ms, TR=7.9 ms, voxel size=1.0×1.0×1.0 mm^3^, FA=15°, FOV=256×256. The structural scan was used for coregistration and anatomical localisation of the functional data obtained from the real-time fMRI neurofeedback data acquisition.

### 2.4 Real-time fMRI procedure

**Fig. 1:**
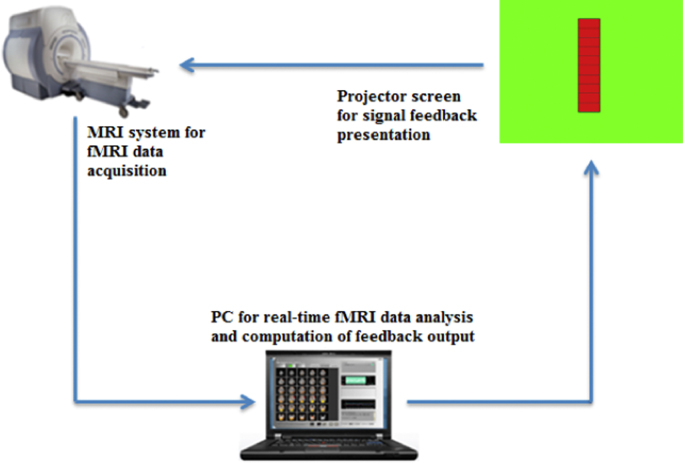
The components of the neurofeedback setup. Data are acquired in the MRI scanner and transferred in real time to an analysis computer, where they are also analysed in real time (for example one frame every 2 s). A translation algorithm then converts the extracted feature of the MRI signal into a command for a feedback signal, which can be displayed to the participant while he or she is in the scanner.

The real-time fMRI neurofeedback platform used for the present study entailed three main stages; the signal acquisition stage, the signal analysis stage and signal feedback stage (see Fig. 1). The signal analysis and signal feedback stages resided on the same computer. The platform is similar to the architecture described in Sokunbi et al. (2014). The first step in the signal acquisition stage was an 11-min localiser run which was used to discriminate the regions of interest (ROIs; FFA and PPA) that was used for subsequent neurofeedback runs. In the localiser run participants passively watched faces, indoor and outdoor scenes as well as animals from a database (provided by Prof Paul Downing, Bangor University). Stimulus blocks were presented in pseudo-randomised order, each block consisting of four images of the same category presented for 1.5 s each. The parameters used for the blood-oxygen level dependent (BOLD) echo planar imaging (EPI) sequence were as follows: TR=2000 ms, TE=35 ms, 30 slices, FA=80, FOV=192×192, matrix size=64×64, slice order=interleaved, inplane resolution=3 mm×3 mm, slice thickness=3 mm, gap thickness=1. In the Localiser run, ROIs were defined from the statistical t maps that resulted from the GLM contrast of Faces>Scenes (FFA) and for Scenes>Faces (PPA). Voxels were included in the target ROIs at an investigator chosen t-value threshold between 2 and 3, with a minimum cluster size of eight voxels.

The real-time imaging software, Turbo BrainVoyager (TBV; Brain Innovation B.V., Maastricht, the Netherlands (Goebel, 2001)) reads in the BOLD EPI images, as they are acquired, via access to a shared datastore. The real-time pre-processing includes correction for head movement (both rotational and translational) and long-term signal drift, and the ongoing analysis implements an incremental general linear model (GLM) with three regressors for the Localiser run (Faces, Scenes and Animals) and a single regressor of ‘self-regulation’ for the NF runs. This incremental GLM was used to compute maps to localise PPA and FFA online.

The neurofeedback runs entailed a signal feedback stage, which was achieved with minimal delay from data acquisition and analysis (less than one TR interval). Raw signal was extracted from defined ROIs for the calculation of instantaneous percent signal change (PSC). The instantaneous PSC was calculated by determining the signal level in the most recently acquired three volumes and comparing that to the mean signal from the preceding baseline block (while additionally accounting for the haemodynamic lag in returning to baseline). For the current experiment the signal from both FFA and PPA was extracted and the difference in PSC calculated for presentation to the participant on a thermometer bar (Fig. 1). A 1% increase of the relative BOLD signal was set as maximum level for the thermometer, with each of the 10 red thermometer blocks corresponding to .1% BOLD signal change. Participants in the NF group were informed about the hemodynamic delay, i.e. that it would take between 4 and 8 s for any changes in brain activity to lead to a change of the BOLD signal and thus the thermometer. The software used for the signal feedback analysis and presentation was PsychoPy (Peirce, 2007).

**Fig. 2:**
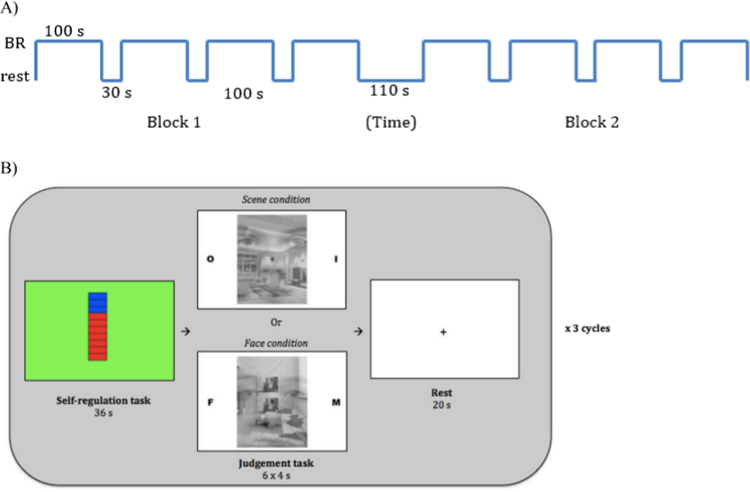
Task design. (A) Binocular rivalry task: Each block consisted of four trials that lasted 100 s each. All trials were separated by a 30 s rest period and both blocks were separated by a rest period of 110 s. Participant are presented with an image of a face superimposed on an image of a scene and indicate via button presses whether the face or scene is experienced as more pronounced. (B) Neurofeedback run with the judgement task: Each run consists of 3 cycles of up-regulation, judgment task (with either gender or indoor/outdoor judgements) and rest.

After a 3 min practice run, six neurofeedback runs of 3 min each were conducted which each consisted of three cycles of the self-regulation task, judgement task and a rest period (see Fig. 2B). During the 36 s self-regulation task participants were asked to increase activation in the PPA as much as possible compared to the activation in the FFA. Participants in both groups were informed about the role of the FFA and PPA in the processing of both real and imagined faces and scenes. It was explained that as a consequence, the most effective way to differentially upregulated activity in FFA was by imagining scenes while refraining from imagining faces. Participants in the NF group were only presented with an increased ‘temperature’ on the thermometer if activation levels in PPA increased more than in FFA. During the 24 s judgement task six picture pairs were presented for 4 s each (see below, Section 2.6). During the 20 s rest period participants were presented with a fixation cross and instructed to count downwards from 99 in steps of three to provide an approximate match for the effort involved in the mental imagery used during the upregulation periods. Respiratory rate and heart rate were recorded throughout the localiser and neurofeedback runs.

Participants in the IM group viewed a static thermometer display in a mock scanner with audio recordings of an echo-planar imaging (EPI) sequence played back to them through speakers.

### 2.5 Binocular rivalry task

Participants completed two blocks of binocular rivalry before (Blocks 1 and 2) and after (Blocks 3 and 4) the neurofeedback scan. The binocular rivalry task was performed in a dark room, and a True3Di™ monitor (Redrover) and polarised glasses were used to present the images. We used an a scene, presented to the left eye, and an image of a face, presented to the right eye. The stimuli were similar to the stimuli used by Tong et al. (1998). The participants used keyboard keys to indicate any switches in the dominant percept, and were asked to keep their blinking rate constant and not to attempt to bias perception of either image. Each block of the binocular rivalry task consisted of four trials that lasted 100 s each. All trials were separated by a 30 s rest period and both blocks were separated by a rest period of 110 s (Fig. 2A). The binocular rivalry measures obtained were the number of periods in which a face (‘*Face_hit*’) or scene (‘*Scene_hit*’) was perceived, the total duration of time that faces and scenes (‘*Face_total,*’ ‘*Scene_total*’) were perceived, and the number of switches between percepts (‘*BR_rate*’).

### 2.6 Judgement (categorisation) task

A 2-alternative forced choice task performed inside the scanner was incorporated as a measure sensitive to any perceptual biases between the house and scene category that were too short-lived to be picked up by the binocular rivalry paradigm (see above, Section 2.5), which was run outside the scanner.

The stimuli were transparent composites of face and scene images (see Fig. 2B). For the face images, nine neutral male and female faces each were selected from the Radboud Faces Database (RaFD; Langner et al., 2010). All faces were cropped to a rectangular shape to eliminate all hair around the head. For the scene images, six images were selected from the Internet for each of three subcategories (landscapes, house interiors and house exteriors). Half of the house exterior images captured the complete front view of the house, the other half captured the front view partially. The 18 pictures in both categories were transformed into grey scale and each face image was paired with one scene image to create 18 picture pairs.

This experiment was performed in two stages. In the first stage we identified, for each observer, the transparency settings that led to an equal frequency of reporting the face and scene that made up each composite pair. The transparency settings required to perceive the two images that made up each picture pair with equal likelihood, were measured for each participant individually via a staircase procedure implemented in PsychoPy (Peirce, 2007). The staircase procedure increased the transparency of the face image benchmarked against a fixed transparency of .5 for the scene image if the face was judged as less prominent. In turn it decreased the transparency when it was judged as more prominent. Transparency changes initially occurred in steps of .1, after two reversals this lowered to .05 and after another two reversals to .01. The staircase procedure finished after at least 15 responses and at least 4 reversals had occurred.

The judgement task comprised two conditions, “face” and “scene.” In the face condition, participants had to decide whether the face was male or female and to make a button press accordingly. In the scene condition, participants made a button press to indicate whether the scene represented an indoor or outdoor scene. The letters flanking the picture pair indicated whether participants had to judge the face or scene and were either an M (male) and F (female), or an I (indoor) and O (outdoor). The side on which these letters were presented was counterbalanced across participants. Participants were asked to respond as quickly and accurately as possible and reaction time (RT) and accuracy were recorded.

Participants performed the judgement task once before the neurofeedback procedure (while the FSPGR sequence was acquired for the NF group, or the sound of a recorded FSPGR sequence played to the IM group). Overall 54 stimuli (three repetitions for each composite stimulus) were presented for each condition (scene and face judgements). They performed the judgement task again during the neurofeedback runs, divided into 18 blocks of six stimuli (three each for the scene and face judgements) after each self-regulation run. All reaction times faster than 200 ms or slower than 3000 ms were classified as incorrect and excluded from the RT analysis. Button presses were recorded via an MRI compatible LumiTouch™ (Photon Control, Canada) response box. Participants were asked to maintain fixation on a fixation cross that was presented in the middle of each composite stimulus.

### 2.7 Eye tracking

Activation changes in the frontal eye fields (FEF) that are related to eye movement have been shown to affect activation in visual areas (Taylor et al., 2007). To control for this potential confound we recorded eye movements with an – MRI compatible eye tracking system (SMI iView X™, SensoMotoric Instruments), which recorded the pupil position of the right eye with a sampling rate of 50 Hz. The Euclidean distance between the origin of the tracker's coordinate system and pupil position was calculated for each sampling point during the self-regulation blocks in order to estimate the total distance moved. These values were correlated with self-regulation performance in the PPA to investigate the relation between activation increments and eye movement.

### 2.8 Offline fMRI data analysis

BrainVoyager QX 2.3 (Brain Innovation, Maastricht, The Netherlands) was used for offline imaging analysis. Standard fMRI pre-processing steps were carried out including motion correction, temporal high pass filtering (2 sine/cosine pairs), spatial smoothing (6 mm FWHM Gaussian filter) and temporal smoothing (3 s FWHM Gaussian filter). All self-regulation runs were aligned to the first volume of the localiser run and then co-registered to the structural image and normalised into the standard Talairach stereotaxic space (Talairach and Tournoux, 1988). Heart rate and respiratory rate measures were included as covariates in the general linear model (GLM). The beta-values and *t*-statistics for the self-regulation predictor in the GLM of the FFA and PPA regions-of-interest were extracted for each run as a measure of self-regulation performance.

Even if PPA activation during upregulation is higher than FFA activation, this does not necessarily imply successful upregulation of the PPA because this area may be intrinsically more active during a variety of tasks. We therefore compared the PPA to FFA difference during neurofeedback with the PPA to FFA difference during the judgement task and required the former difference to be higher than the latter before we would assume effects of genuine upregulation. To perform this, beta values were derived from the GLM. Then, for PPA and FFA during both judgement and neurofeedback conditions, the beta values from the GLM were z-transformed using the following equation:

z Value for run=(beta value for run−average beta value for all runs of this condition)/standard deviation for all runs of this condition.

Finally we calculated the differences in z values for PPA and FFA for the NF and judgement predictors (NFDIFF=PPA_NF_−FFA_NF_; JDGDIFF=PPA_J_−FFA_J_) for each participant, and group differences were tested using a one-tailed paired *t*-test to test the hypothesis of genuine upregulation during neurofeedback.

### 2.9 Exposure task

In order to ascertain whether any perceptual changes on the BR or judgement tasks would be specific to the neurofeedback procedure we conducted a control procedure involving the same assessments (judgement and rivalry task) and dependent measures as the neurofeedback experiment but exposure to visual stimuli of one category (in this case faces) instead of neurofeedback as independent variable. Ten healthy students participated in this experiment (see Section 2.1). In order to induce perceptual biases we foveally presented face stimuli subtending 7.4 (horizontally) and 9° (vertically) visual angle (duration: 3 s, inter stimulus interval: .5 s) for 24 min. In total 9 face pictures (drawn from a set of 18 different faces, including the one used in the binocular rivalry task) were shown. Before and after the exposure task participants performed the same judgement and binocular rivalry task as the participants in the main study.

## 3 Results

### 3.1 Imaging

**Fig. 3:**
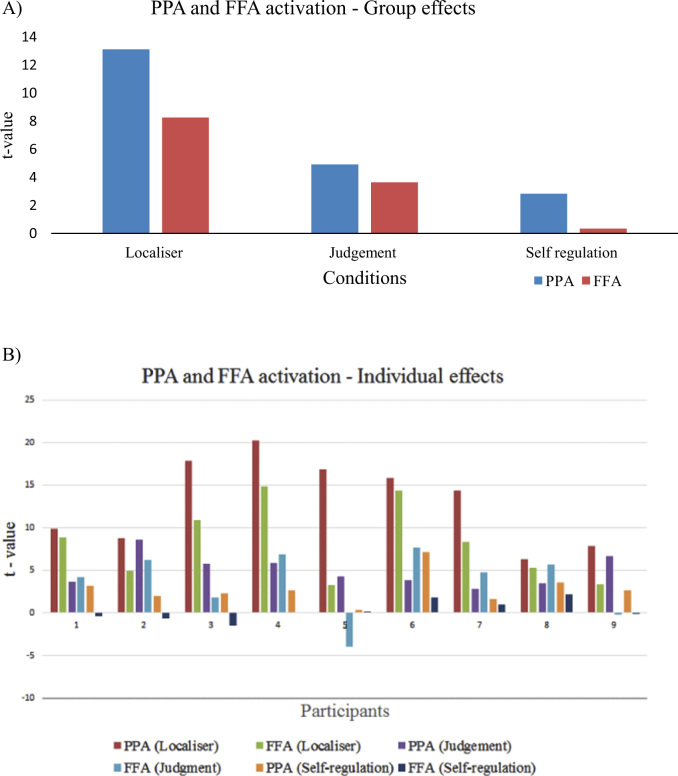
Self-regulation of PPA vs. FFA. (A) Activation levels in the parahippocampal place area (PPA) and fusiform face area (FFA) in the three scanning conditions (localiser, judgement task, self-regulation), represented by the t-statistic of the respective predictor and averaged over all runs. As expected, real-stimuli resulted in stronger activation than imagined stimuli. (B) Individual data for the nine participants. These show that, regardless of interindividual variability in activation levels, participants were generally able to obtain a higher activation level in the PPA (orange bars) than in the FFA (dark blue bars) during the self-regulation condition, as per instruction.

All participants were able to carry out the self-regulation task successfully: averaged over six runs all participants showed higher activation in the PPA than in the FFA (one sample *t*-test, *t*(8)=4.670, *p*=.002; Fig. 3).

**Fig. 4:**
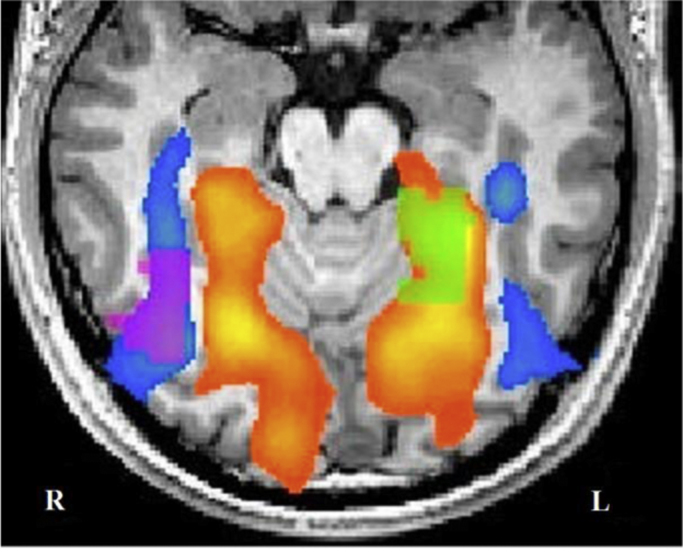
Localisation of the target areas. Results from an individual localiser run, showing a contrast map for face – (in cold colours) vs. scene – (in hot colours) related activation. Target areas selected for neurofeedback in the right FFA (purple) and left PPA (green) are indicated. Image presented in radiological convention (right side of the brain is left side of the image).

PPA target areas were significantly larger than FFA target areas (*t*(16)=3.862, *p*=.001; Table 1; see Fig. 4 for location of PPA and FFA as identified by the localiser run). It could be argued that individual differences in self-regulation of the PPA and FFA were mediated by differences in target size. We found evidence in support of this by showing a significant correlation between ROI size and self-regulation ability, defined as the absolute *t*-statistic of the upregulation predictor (*r*=.505, *p*=.033). Furthermore, ROI size correlated negatively with the intraindividual variability of self-regulation (standard deviation of activation levels across runs in the PPA) (r=−.7266, p=.027).

In general, PPA activation during self-regulation was less pronounced than that induced by physically presented stimuli during the judgement task, but it was much higher relative to FFA activation (Fig. 3). We confirmed a genuine upregulation effect during NF by showing a significant difference between NFDIFF and JDGDIFF (one sample *t*-test, t(8)=1.9, p=.047); that is, the PPA over FFA activity was significantly higher during neurofeedback than during judgement blocks.

**Fig. 5:**
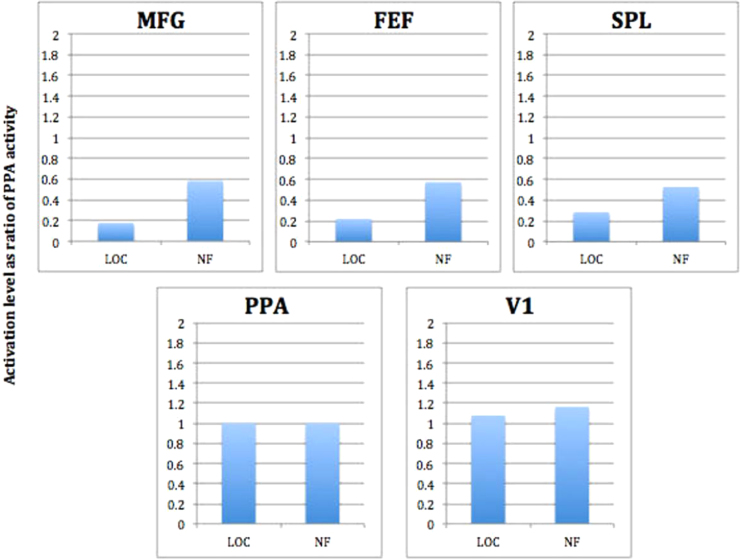
Activation patterns in five areas activated during both the localiser (LOC) run and neurofeedback (NF) run. Activation levels (as ratio of PPA activity) are shown for the Middle frontal gyrus (MFG), Frontal eye fields (FEF), Superior parietal lobule (SPL) and the Visual cortex (V1). Areas with a larger synaptic distance from the primary visual cortex (V1) seem to be activated relatively more during the neurofeedback than the localiser runs.

Our next aim was to determine patterns of whole-brain activation accompanying successful self-regulation training of higher visual areas. A previous functional imaging study adopting a visual motion imagery paradigm found that the larger the synaptic distance between an activated area and V1, the greater the activation of that area during imagery (Goebel et al., 1998). To test if our data showed a similar pattern, five areas at different levels of the visual processing hierarchy were selected that were activated during both the presentation of scenes in the localiser run and during the imagery of scenes in the self-regulation runs (Table 2). Beta values from those five areas (incorporating all activated voxels within a 2×2×2 cm^3^ box centred over the peak voxels in the left and right hemisphere) were extracted and averaged over both hemispheres. The activation level in the PPA was set as 1 and the activity in other areas was calculated as a ratio of PPA activation because the durations of the localiser and self-regulation condition were different (Fig. 5). As a main result, we found the predicted pattern of higher relative activation in prefrontal and parietal areas during the NF compared to the localiser condition.

### 3.2 Behavioural results: judgement task

A two-way MANOVA with the factors *Group* (NF/IM) and *Time* (Baseline/Scan) was conducted with the dependent variables ‘Faces RT’, ‘Scene RT’, ‘Faces Accuracy’ and ‘Scene Accuracy’. The MANOVA did not yield a significant interaction (*F* [4,25]=.296, *p*=.878) but returned a significant effect of *Group* (*F* [4,25]=2.942, *p*=.04) and a marginally significant effect of *Time* (*F* [4,25]=2.566, *p*=.063). The main effect of *Group* was caused by higher ‘Scene Accuracy’ scores in the NF than IM group at both time points (*F* [1,28]=6.703, *p*=.015) (Table 3). The NF manipulation thus had no effect on performance of the judgement task, as evidenced by the lack of significant interaction between *Group* and *Time*.

### 3.3 Behavioural results: binocular rivalry task

We conducted a two-way MANOVA with the factors *Group* (NF/IM) and *Time* (pre/post intervention) and the dependent variables “Face Hit’, ‘Scene Hit’, ‘Face Total’, ‘Scene Total’, and ‘BRrate’. We did not find any significant differences between both groups at baseline for any of the binocular rivalry measures (all ps>.1). We did not find a significant interaction between *Group* and *Time* (*Pillai's* F [5,56]=.477, p=.41). A main effect was found for Group (Pillai's F [5,56]=3.453, p=.009) but not for Time (*Pillai's* F=[5,56]=.317, p=.901). Post-hoc tests showed that the significant effect of Group was mediated by group differences in the variables ‘Scene Total’ (smaller in the NF group) (F [1,56]=15.166, p=.000; and ‘Face Total’ (smaller in the IM group) (F [1,56]=11.1677, p=.001). Thus, as in the judgement task (reported above, Section 3.2) we found no effect of the experimental manipulation on the perceptual measures, as evidenced by lack of interaction between *Group* and *Time*.

### 3.4 Eye tracking

We had usable eye tracking data from four participants; no significant correlations were found between total eye movements and self-regulation ability in the PPA (*r*=.093, *p*=.690). The increase in activation in the PPA during the self-regulation task could thus not be attributed to eye movements in these participants.

### 3.5 Correlations with psychometric measures

There were no significant correlations between self-regulation ability and TCQ or TCAQ scores (all *p*s>.6).

### 3.6 Behavioural results: exposure task

This task assessed whether intensive exposure to stimuli of one category (faces) would induce perceptual biases in the binocular rivalry and judgement tasks. These two tasks were conducted before and after the manipulation. No significant effect of exposure was observed.

A 2×2 repeated measures ANOVA was conducted to compare the *duration of predominance* of 'face’ and 'scene’ in the pre- and post-exposure session of the binocular rivalry task. Our main interest was in any interaction between percept and time, which would have indicated a modulation of perception by the experimental manipulation. However, we did not find a significant interaction between the two sessions (pre/post) and the stimuli (face/scene) (*F* [1,9]=1.460, *p*=.258). A main effect was found for the different percepts (face (*M*=267.36s, *SD*=94.06) and scene (*M*=445.87s, *SD*=48.6), *F* [1,9]=22.410, *p*=.001) but no significant difference between pre- and post-exposure sessions (*F* [1,9]=1.901, *p*=.201).

A repeated measures ANOVA was also conducted to compare the *number of face and scene periods* in pre- and post-exposure sessions. There was a significant main effect of stimulus (F [1,9]=22.410, p=.001) but no significant interaction of stimulus and time (F [1,9]=1.620, p=.235) indicating that the intervention had no effect on the number of periods during which either scenes or faces were perceived.

## 4 Discussion

This study confirmed that it is possible to exercise control over the activity in visual processing areas via fMRI-based neurofeedback training. We confirm recent reports of the feasibility of upregulating the PPA (Harmelech et al., 2015, Ekanayake et al., 2013). We furthermore demonstrate that differential feedback can be used to exercise control over visual areas (Fig. 3) and that the upregulation we observed is due to specific mental processes rather than differences in eye movements across conditions, increases in whole brain activation, or basic physiological (cardiovascular) processes. Similar to non-feedback imagery tasks, imagery-based upregulation of higher visual areas seems to be supported by activation of prefrontal and superior parietal areas known to be involved in the generation of visuospatial mental images (Goebel et al., 1998, Trojano et al., 2000; Formisano et al., 2002; Sack et al., 2002).

One intriguing finding is the relationship between target area size and self-regulation ability. We provide preliminary evidence that larger target areas lead to a more temporally stable estimate of cortical activity in much the same way as spatial smoothing leads to increased signal to noise in regular fMRI. This information is useful for the design of future neurofeedback protocols although target region size has to be balanced against the desired functional specificity.

We had hypothesised that the NF group would show larger perceptual changes after mental imagery combined with neurofeedback than the IM group after mental imagery alone, since the PPA over FFA neurofeedback signal was intended to help fine-tune the participants’ strategies towards imagery of scenes/places rather than faces. The results of the judgement task however suggest that there was no immediate perceptual effect of neurofeedback training. The absence of a significant *Group*×*Time* interaction may be partly explained by a ceiling effect as the low number of incorrect responses in the scene condition left little room for improvement following training, although this would not apply to the more difficult face condition. While the results show that participants became faster in judging faces and scenes, this improvement was comparable in both groups; since this improvement was found for both faces and scenes it is likely that the underlying cause is general learning rather than a behavioural change induced by mental imagery, or, in case of the NF group, the self-regulation training.

Contrary to the findings of Ekanayake et al. (2013), in the present study, neurofeedback training of the upregulation of PPA activity had no effect on bistable perception. Several factors may have contributed to this outcome. First, even though the PPA and FFA are involved in the processing of scenes and faces, and it has been shown that activation in the FFA and PPA reflects the dominant percept in a binocular rivalry task (Tong et al., 1998), other brain areas may control the perceptual state. Indeed, it has been suggested that rivalry may be resolved at a lower level of visual processing (Tong et al., 1998), and studies using TMS or caloric stimulation to interfere with binocular rivalry perception suggest that the parietal cortex may be the primary site controlling the temporal dynamics of binocular rivalry (Miller et al., 2000, Carmel et al., 2010). A second explanation is that the overall duration of our neurofeedback training may have been too short. Scharnowski et al. (2012) presented feedback for a total of 49 min, and Shibata et al. (2011) conducted either five or ten sessions so participants executed either a total of 81 or 162 min of self-regulation, much longer than the total 15 min of neurofeedback of the current study. However, even more intense NF training may not modulate visual areas sufficiently to obtain lasting perceptual effects. For example, Scharnowski et al. (2012) only found enhancement when the perceptual sensitivity task was carried synchronously with the neurofeedback training. Similarly any neurofeedback-induced changes of bistable perception may be too short-lived to be detected after the end of the scanning session and neurofeedback during the presentation of the bistable stimulus may be required for any perceptual changes to occur.

In conclusion, the results from the current proof-of-concept study show the feasibility of differential self-regulation of a higher order visual processing area in healthy volunteers. However, the ability to upregulate the PPA over the FFA did not result in concomitant changes in perceptual biases, as assessed by the judgement task with transparent stimuli and the binocular rivalry paradigm with bistable stimuli.

## Figures and Tables

**Table 1:** Results of the localiser run (identification of PPA and FFA ROIs) for individual participants (2 columns).

	**ROI details**			
**PPA**		**FFA**	
**Participant**	*Nr of voxels*	*TAL coordinates*	*Nr of voxels*	*TAL coordinates*
*1*	2945	25, −42, −15	2192	48, −59, −16
*2*	4245	23, −38, −13	940	35, −46, −21
*3*	3409	−24, −43, −13	3849	42, −62, −12
*4*	4481	−23, −52, −16	1362	42, −62, −19
*5*	1979	−22, −47, −9	1353	44, −65, −10
*6*	4000	−22, −45, −13	594	36, −47, −19
*7*	3447	−23, −47, −13	1827	42, −64, −14
*8*	2206	−25, −4, −11	1446	41, −62, −17
*9*	5429	−21, −55, −12	1819	−52, −44, −10

**Table 2:** Areas of overlapping activation in the localiser and neurofeedback runs with their respective centres of mass (1.5 columns).

	**TAL coordinates**	
	*Localiser run*	*Neurofeedback runs*

**V**1	L: −10, −89, −9	L: −12, −94, −8
R: 11, −89, −6	R: 8, −95, −8
		
**PPA**	L: −26, −53, −13	L: −27, −42, −11
R: 26, −50, −14	R: 24, −38, −11
		
**SPL**	L: −29, −58, 38	L: −23, −63, 38
R: 25, −63, 36	R: 26, −57, 38
		
**FEF**	L: −39, −15, 45	L: −42, −6, 45
R: 40, −11, 40	R: 41, −9, 43
		
**MFG**	L: −38, 9, 26	L: −47, 16, 28
R: 35, 18, 24	R: 45, 20, 25

**Table 3:** Performance on the judgement task (2 columns).

	**Face task baseline**	**Face task scan**	**Scene task baseline**	**Scene task scan**
**Incorrect Responses (NF)**	**19.6 (1.782)**	**18.3 (1.236)**	**1.5 (.463)**	**0.9 (.398)**
**Incorrect Responses (IM)**	**19.1 (.895)**	**18.9 (1.260)**	**3.4 (.981)**	**2.3 (.491)**
**RT (NF)**	**1614 (45.36)**	**1465 (57.00)**	**1350 (48.63)**	**1212 (43.33)**
**RT (IM)**	**1689 (50.14)**	**1444 (51.30)**	**1376 (49.46)**	**1156 (41.90)**

Accuracy is represented by the counts of incorrect responses (out of 54 trials in each condition). RT is presented in milliseconds. Values in parentheses are standard errors of the mean.
